# Study protocol for the development and validation of a questionnaire evaluating predisposition to immunosuppressant medication non-adherence of kidney pre-transplant patients. The KATITA project

**DOI:** 10.1371/journal.pone.0305953

**Published:** 2024-06-25

**Authors:** Luana Cristina Lins de Medeiros Oliveira, Rand Randall Martins, Antonio Gouveia Oliveira

**Affiliations:** 1 Graduate Program in Pharmaceutical Sciences, Centro de Ciências da Saúde, Universidade Federal do Rio Grande do Norte, Natal, RN, Brazil; 2 Clinical Pharmacy Unit, Onofre Lopes University Hospital, Centro de Ciências da Saúde, Universidade Federal do Rio Grande do Norte, Natal, RN, Brazil; 3 Department of Pharmacy, Centro de Ciências da Saúde, Universidade Federal do Rio Grande do Norte, Natal, RN, Brazil; Medical University of Gdansk, POLAND

## Abstract

Non-adherence to immunosuppressive medication after kidney transplant is an important cause of graft rejection and loss. Approaches to minimization of non-adherence have focused on the identification of episodes of medication non-adherence, but by then irreparable harm to the graft may already have occurred, and a more effective approach would be to adopt preventive measures in patients who may have difficulty in adhering to medication. The aim of this study protocol is to develop and validate a clinical questionnaire for assessing, in kidney transplant candidate patients in the pre-transplant setting, the predisposition to non-adherence to immunosuppressive medication. In this multicenter, prospective study, a pilot questionnaire in Brazilian Portuguese language, composed of Likert-scaled statements expressing patients’ beliefs, behaviors and barriers regarding medication taking will be assembled from a literature review, from focus groups, and an expert panel. The pilot questionnaire will be administered to a minimum of 300 patients in kidney transplant waiting lists and exploratory factor analysis will be used for development of the definitive questionnaire. A random subsample of a minimum of 60 patients will have the scale re-administered after one month for evaluation of test-retest reliability. A multicenter, external validation study will include 364 kidney transplant candidates who will be evaluated immediately before surgery and at months 3, 6 and 12 post-transplant for assessment of concurrent validity, by comparison with two scales that assess medication non-adherence, and for determination of predictive validity using a triangulation method for assessment of medication non-adherence. Structural validity will be assessed with confirmatory factor analysis using structural equation modeling. Cross-cultural generalizability and validity will be assessed by a multicenter study, in which a translation of the scale to another language will be administered to kidney transplant candidate patients from a different culture, with a subsample being selected for test-retest. This study will be conducted in Spain with a Spanish translation of the scale.

## Introduction

Chronic Kidney Disease (CKD) was defined by the National Kidney Foundation, in its document Kidney Disease Outcomes Quality Initiative, as kidney damage present for a period equal to or greater than three months, characterized by structural or functional abnormalities of the kidney, with or without a decrease in the glomerular filtration rate, manifested by histopathological abnormalities or markers of kidney damage, including blood or urinary changes, or in imaging tests [1]. CKD is an important medical and public health problem, and its prevalence is increasing worldwide [2, 3]. It is estimated that more than 10% of the global population suffers from CKD [4]. In Brazil, according to the 2016 census of the Brazilian Society of Nephrology, it is estimated that there are 122,825 patients in dialysis programs with CKD in its most advanced stage [5]. This number represents an increase of 31,500 patients in the last 5 years (91,314 in 2011) [5].

Renal replacement therapies such as hemodialysis, peritoneal dialysis and kidney transplantation are forms of treatment for patients with stage 5 CKD, in the functional failure stage [3, 6]. Kidney Transplantation is currently considered the best therapeutic option for patients with CKD in its most advanced stage, both from medical, social and economic point of views [7–9]. It increases quality-adjusted life years by five times and is more cost-effective when compared to dialysis.

Although superior to dialysis treatment, kidney transplantation involves the inherent risk of rejection and/or graft failure that incurs costly hospitalizations, laboratory tests and anti-rejection treatments that are associated with poor patient outcomes [3, 10–14]. To minimize the risk of rejection, recipient patients are placed on lifelong regimens of immunosuppressive drug treatments [15] and are monitored for signs of rejection [3]. A combination of two or three different immunosuppressants are taken on a long-term basis to prevent rejection. However, as with all medications, patients may experience adverse reactions and these may include an increased risk of infections, diabetes, increased susceptibility to certain cancers, increased blood pressure and weight gain [12]. It is also true that the immunosuppression process can increase the risk of comorbidities. Despite the nuances of the medication, adherence to the immunosuppressant regimen is vital to providing the grafted kidney with the best chance of survival and function after kidney transplantation [6]. Graft survival after kidney transplantation emphasizes adherence to immunosuppressive therapy, [11, 12, 16] as well as adherence to other prescribed medications, which are important to ensure control of the comorbidities that commonly accompany patients with end-stage CKD and thus not compromising the health of the patient and the transplanted kidney [7].

However, despite a long and frustrating time on the waiting list following a successful transplant, many patients develop problems with adherence to their immunosuppressive medications [7, 16]. Not taking prescribed immunosuppressive drugs (defined as taking drugs <95.1% of days) is associated with a 60% increased risk of kidney transplant failure and premature death [11, 17]. Adherence in this context refers to the “extent to which the patient’s behavior matches agreed prescriber recommendations” [6], and non-adherence (NA) as “deviation from the prescribed drug regimen sufficient to negatively influence the effect” [18]. In a broader context, adherence is defined by the World Health Organization as “the extent to which a person’s behavior–taking medication, following a diet and/or carrying out lifestyle changes–corresponds to the agreed recommendations of a health professional” [19]. Non-adherence to immunosuppressive medication is an important risk factor for unfavorable post-transplant clinical outcomes, reducing patient survival and causing waste of health resources [20]. However, it remains a common problem in this patient population and has been identified as the second common cause of late graft failure in kidney transplant patients. In 2011, Suárez et al. [21] estimated that the non-adherence rate is between 20% and 54% and that the lack of compliance can contribute to 20% and 16% of graft loss. A recent meta-analysis revealed that the magnitude of NA for immunosuppressants in kidney transplant recipients was as high as 35.6 cases per 100 patient-years. The incidence of NA among kidney transplant recipients was significantly higher than that of the general population of solid organ transplant recipients, which was 22.6 cases per 100 patient-years. That study estimated that 15% to 60% of late acute rejections and 5% to 36% of graft losses were associated with NA in kidney transplant patients [18, 22].

The World Health Organization categorizes non-adherence as a multidimensional phenomenon, determined by 5 factors: economic and structural, complexity of the therapeutic regimen, satisfaction with care, standard of care and patient factors [19, 23]. Within patient factors, the literature has mentioned that adherence is associated with 3 dimensions: behavior, beliefs and barriers. Thus, there is a need for easy-to-use scales that correctly measure the 3 dimensions associated with medication use that influence adherence: medication-taking behavior, barriers to adherence, and patients’ beliefs about medication.

A 2013 systematic review of adherence scales evaluated sixty articles that described or evaluated 43 scales [24]. These scales included items that elucidated information about the patient’s medication-taking behavior and/or attempts to identify barriers to good medication-taking behavior or beliefs associated with adherence, and were categorized into five groups based on the information they were intended to elucidate. Each group had 2 to 3 scales considered the most representative based on the number of correlated and developmental studies. The scales in group 1 encompassed information only on medication-taking behavior, in which the MARS-5 scale was the highlight, consisting of only 5 questions and scored on the Likert scale; the scales of group 2 sought information on medication-taking behavior and barriers to adherence, and the most representative were the ASK-20 (Adherence Starts with Knowledge-20), which is a scale consisting of 20 questions validated in patients with asthma, diabetes or depression, the RAM (Reported Adherence to Medicine), which assesses beliefs about medicines for general and personal use and was validated with patients with chronic diseases (asthmatic, diabetic, psychiatric and undergoing dialysis) and the MMAS (Morisky Medication Adherence Scale) which examines the psychometric properties in patients with hypertension, consisting of 8 questions; the scales of group 3 assess adherence barriers and the most representative was the MAQ (Medication Adherence Questionnaire); group 4 seeks information only on beliefs associated with adherence and is represented by the RAM (Reported Adherence to Medicine); those in group 5 look for information about barriers and beliefs associated with adherence, they are: CQR (Compliance Questionnaire Rheumatology) and MARS.

All scales identified in this systematic review were intended to identify patients who, retrospectively, show insufficient adherence to treatment. There has been a large effort in the search for risk factors of NA in kidney transplant patients but, surprisingly, such efforts have not been followed by attempts to develop instruments for assessing the risk of NA in kidney pre-transplant patients. To the best of our knowledge, only two NA risk stratification tools have been published [25, 26] but intended to be applied only after transplant. Given the high number of kidney transplants performed, the great importance of adherence by patients with immunosuppressants after kidney transplantation in the results of kidney transplantation, the high cost for the health system with anti-rejection treatment with risk, including, of returning to hemodialysis and, as a last resort, a retransplantation, and the lack of a clinical questionnaire that assesses the predisposition to adherence to immunosuppressants to be applied in kidney pre-transplantation patients, it is clear that this community needs effective tools to identify patients at risk for NA, before undergoing kidney transplantation, so that actions can be taken to monitor the treatment, prevent non-adherence and motivate the patient to reduce the risk of adverse outcomes arising from non-adherence. In clinical practice, such tool could be used for assessment of medication adherence of kidney transplant candidates in the pre-transplantation setting, which, in combination with an evaluation of risk factors of NA and adherence barriers, could guide personalized adherence plans including appropriate education, counseling and post-transplant surveillance. In clinical research, that tool could be useful in the evaluation of interventions directed to improving patients’ beliefs and behaviors towards medication, and in the selection of prescription-complying patients for inclusion in randomized clinical trials of immunosuppressants.

The Kidney AlloTransplant Immunosuppressant Therapy Adherence (KATITA) project was set up to address the unmet medical need for a tool assessing the predisposition to immunosuppressant medication non-adherence of kidney transplant candidate patients, in the pre-transplant setting. The project aims at the full development of a validated KATITA scale and is divided into four sequential phases: development of a psychometric scale, internal validation study, external validation study, and validation study of a cross-cultural adaptation to a different language.

## Materials and methods

### Objectives

The objectives of each project phase are as follows: First phase, development of a psychometric scale: 1) to develop a comprehensive item pool for KATITA; 2) to identify the psychometric dimensions of medication non-adherence in the pre-transplant setting; 3) to select the relevant items composing the final KATITA questionnaire. Second phase, internal validation study: 1) to evaluate internal consistency reliability of KATITA; 2) to evaluate test-retest reliability of KATITA; 3) to evaluate construct validity by assessing convergent validity. Third phase, external validation study: 1) to evaluate criterion validity by assessing concurrent validity and predictive validity; 2) to evaluate the scale structural validity. Fourth phase, transcultural adaptation to a different language: 1) to translate KATITA to a different language using the translation-back translation method; 2) to evaluate reliability of the KATITA translation using internal consistency and test-retest reliability methods.

The study protocol was approved by the Research Ethics Committee of the coordinating center at Hospital Universitario Onofre Lopes in Natal-RN, Brazil (authorization number 3.179.920 of March 1, 2019). All four study phases are considered minimal risk research, no experimental interventions will be performed on patients, and no additional data will be collected beyond the necessary for usual routine care. Approval will be obtained from the Institutional Review Boards of every collaborating center and signed informed consent will be obtained from all participating patients.

### Scale development

**Fig 1:**
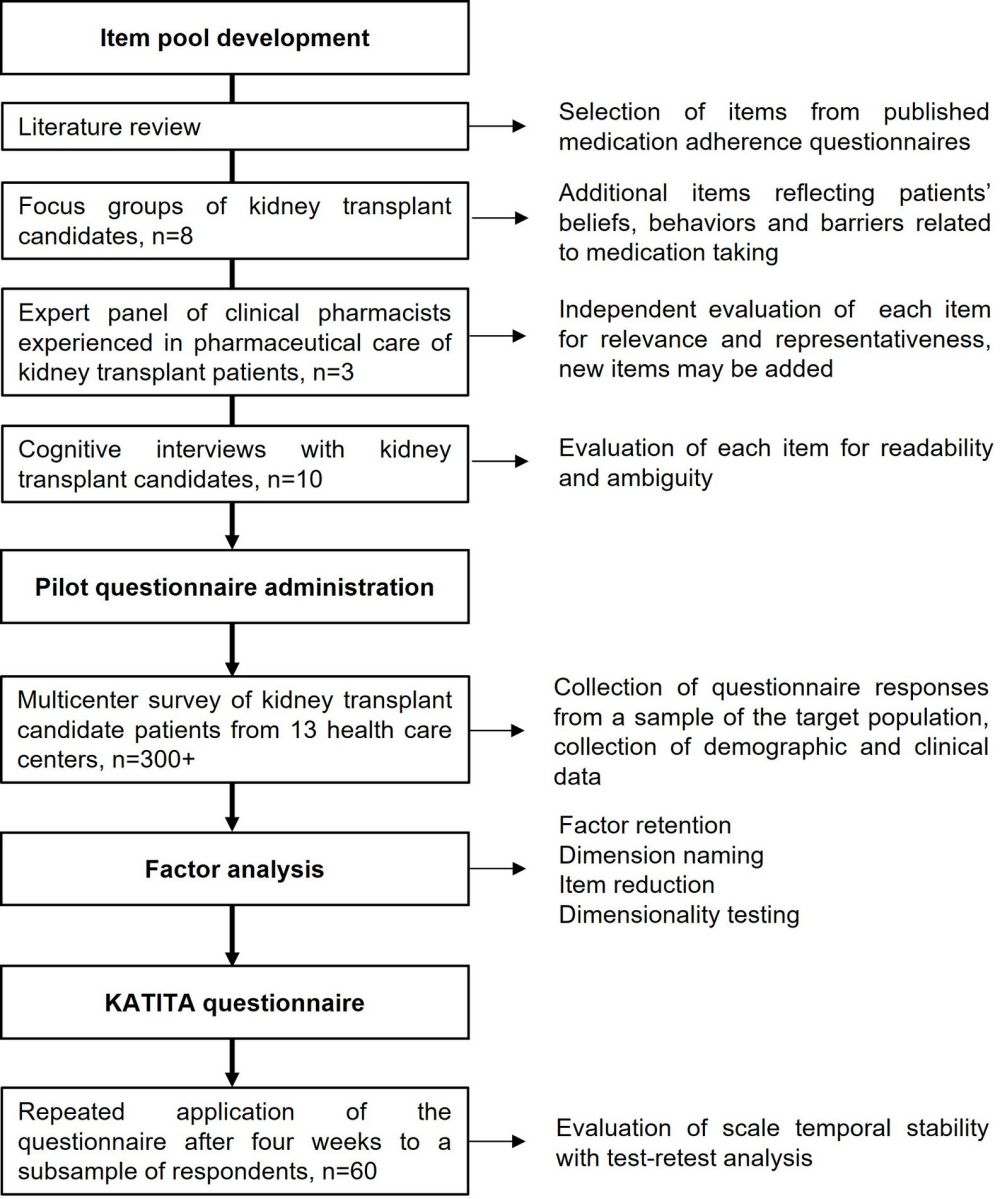
Flowchart of the scale development process. The chief steps are shown in boxes, with arrows pointing to the main objectives of each step.

A flowchart of the scale development process is shown in Fig 1, illustrating the steps of the scale development process, the objectives of each step, and the sample size planned for each step.

#### Item pool development

Once the construct is clearly identified, based on the definitions of medication non-adherence presented above, the first step in scale development will be item generation, the identification of the appropriate questionnaire questions, which will combine a deductive and an inductive method. The former will consist of a search for medication adherence scales from bibliographic databases. Statements from those scales related to patients’ medication-taking behaviors, to barriers to good medication-taking behavior, or to beliefs associated with medication adherence will be selected for inclusion in a preliminary item pool of potential statements for the KATITA scale. The inductive method will consist of a focus group of consecutive patients attending the nephrology clinic of the coordinating center, of both sexes, over 18-years-old, who are in the kidney transplant waiting list, moderated by a psychologist experienced in kidney transplant patients. The patients will be asked to review and reformulate or rephrase the previous selected items, and add new ones reflecting their experiences and thoughts on medication taking. The focus group sessions duration should not exceed 60 minutes and will be repeated until saturation occurs, that is, when no additional concepts are elicited in two consecutive sessions. The focus group sessions will be recorded and the transcripts will be reviewed by the study investigators, who will select all the statements that may reflect patients’ attitudes, behaviors, beliefs and barriers to medication adherence.

The next task will be the assessment of content validity by a panel of three clinical pharmacists with extensive experience in the pharmaceutical care of kidney transplant patients. These expert judges, based on their experience in interactions with this population during pharmacotherapeutic consultations, will independently review each item to decide, through consensus, whether it should be eliminated due to irrelevancy or unrelatedness to medication non-adherence. The panel will also review the wording of each item for complexity and ambiguity, and may generate new questionnaire items.

The resulting item pool will be submitted to cognitive interviews with a small sample of patients selected with the same criteria as those of the focus groups but who had not participated in the focus group, to determine whether the intended meaning of each item is fully perceived by the respondents. In this process, patients will respond to each item and then asked to explain why they responded that way [27]. This process will culminate in a set of items that will be used for the construction of the KATITA questionnaire through the statistical analysis of the responses of a random sample of target subjects to each item.

#### Pilot questionnaire administration

Thirteen healthcare centers in three capitals of northeastern Brazil states, of which seven are hemodialysis centers and six are tertiary care hospitals performing kidney transplant, will be included and will enroll consecutive patients over 18 years of age, of both sexes, who are in waiting lists for kidney transplant and are able to read and communicate, excluding patients who are candidates for kidney retransplant. The patients will be asked to self-rate each item in a 5-point Likert scale, according to their level of agreement with the presented statement, from 0 (strongly disagree) to 4 (strongly agree). Demographic data will be collected (age, sex, ethnicity, level of education, social support), as well as clinical data related to kidney disease (current dialysis, time on dialysis, etiology of kidney disease and number of blood transfusions). A subsample of the patients will be asked to repeat the administration of the questionnaire after six weeks for assessment of the scale temporal stability through test-retest analysis. These will be consecutive patients who had responded to the questionnaire.

#### Factor analysis

**Fig 2:**
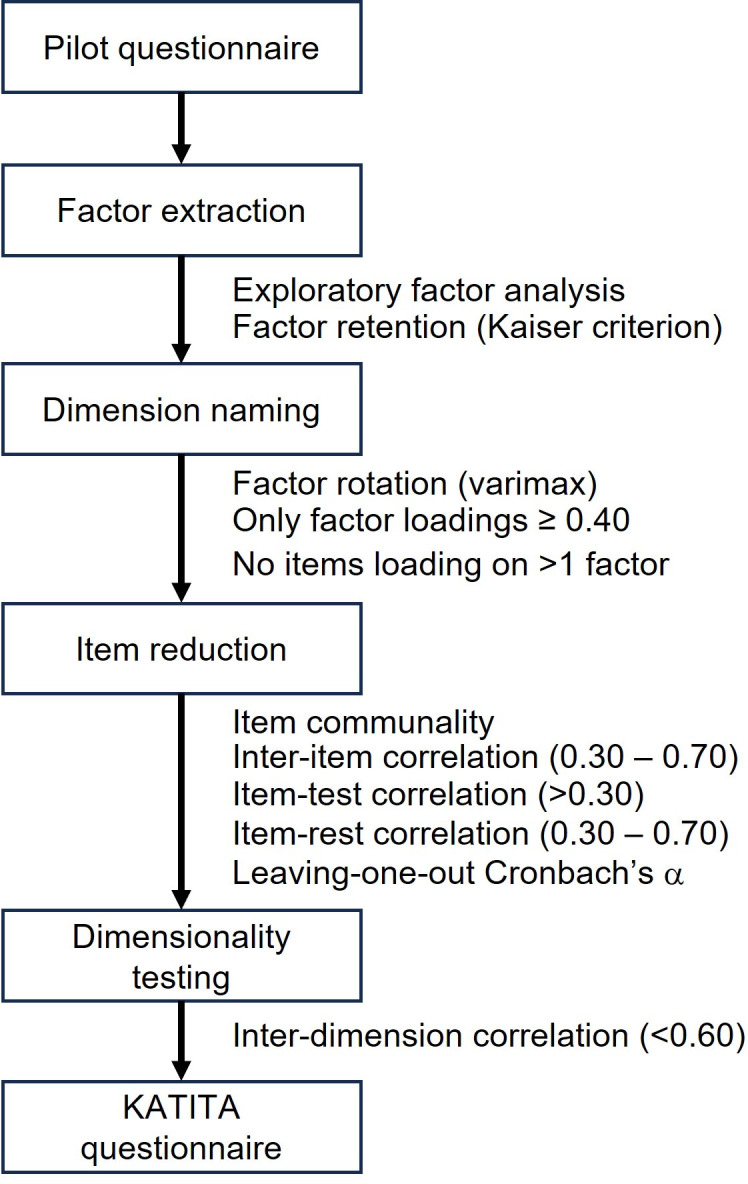
Overview of the steps required for development of the KATITA scale from data obtained with the pilot questionnaire.

The collected data will be analyzed by exploratory factor analysis for factor extraction and subsequent item selection, a process that will result in the final KATITA questionnaire. Fig 2 shows the steps required for the development of the final KATITA questionnaire from the data collected with the pilot questionnaire. Factor analysis is a statistical method widely used for the discovery of unobserved variables, which are called factors, that determined the expression of a set of variables that can be measured. These hidden factors represent dimensions, or facets, of the construct of interest and the meanings of the factors are defined by the investigator, guided by the pattern of relationships between the observed variables and each factor. Factor analysis fits as many factors as there are items in a questionnaire to the empirically obtained dataset, and two important statistics are computed, the factor loadings and the eigenvalues. The former represent the correlation between a questionnaire item and a factor, the latter a measure of the variance in all questionnaire items that is explained by a factor. These statistics will help refining the questionnaire. The eigenvalues are used to select which factors have a significant correlation with the construct of interest, by retaining only those factors with an eigenvalue greater than 1, the so-called Kaiser criterion.

As the dimensions are then reduced to a small number of factors, a better fit to the data can be achieved by positioning only the few retained factors and disregarding the remaining. This is called factor rotation and usually results in higher factor loadings of those items that loaded high on a factor, while those that loaded low will then have loads near zero. The new factor loadings are then used to select the items that are significantly correlated with each factor, retaining only the items with factor loadings above a certain value. This procedure usually causes each item to be associated to at most one factor, making it easier to understand which facet of the construct each factor is measuring. Factor rotation can be orthogonal or oblique, depending on whether the factors are constrained to be at right angles of each other, or free to be placed at the angles that provide the best fit to the data. Although an orthogonal rotation may not fit the data as closely as an oblique rotation, it has the advantage that the factors remain uncorrelated and, therefore, a global scale score can be obtained from the sum of subscale scores, which is not possible after an oblique rotation.

Before proceeding to reliability and internal validity evaluation, item analysis will be conducted to refine the questionnaire. Irrelevant and redundant items do inflate statistics of reliability and therefore should be removed from the questionnaire according to the extent of observed correlations of each item with other items in the scale and with the scale scores [28].

### Internal validity

After these procedures, the final version of the KATITA questionnaire will be obtained and the next step will be validity assessment. Briefly, the complete evaluation of a clinical questionnaire requires proof of reliability, validity, sensitivity, and generalizability. Reliability is assessed by internal consistency reliability and test-retest reliability. Validity is evaluated for two main types, construct validity and criterion validity. Construct validity has two components, content validity and convergent validity, and criterion validity also has two components, concurrent validity and discriminant validity.

Cronbach’s alpha is an indicator of internal consistency reliability of a scale, a measure of the correlation of the items in a questionnaire: if all items are measuring the same construct, it will have the value 1; if all are measuring different constructs, it will be zero. Test-retest reliability is established by showing that a patient’s response to a questionnaire item has a high degree of agreement between two applications of the questionnaire separated by a time lag of sufficient length to minimize recall.

Convergent validity, the second component of construct validity, is shown by a high correlation between the questionnaire scores and other measures predicted by theory. Ideally, convergent validity should be demonstrated by high correlations between the scale and validated scales measuring the same or closely related constructs. However, at the present time this will not be possible due to the inexistence of scales measuring tendency to medication non-adherence before the initiation of therapy. Therefore, in this case convergent validity will be assessed with differentiation by known groups, an accepted method of convergent validity analysis where the distributions of scale scores are compared between levels of known predictors of medication non-adherence. If results are within acceptable values, the scale development will proceed to the external validation phase.

### External validity

**Fig 3:**
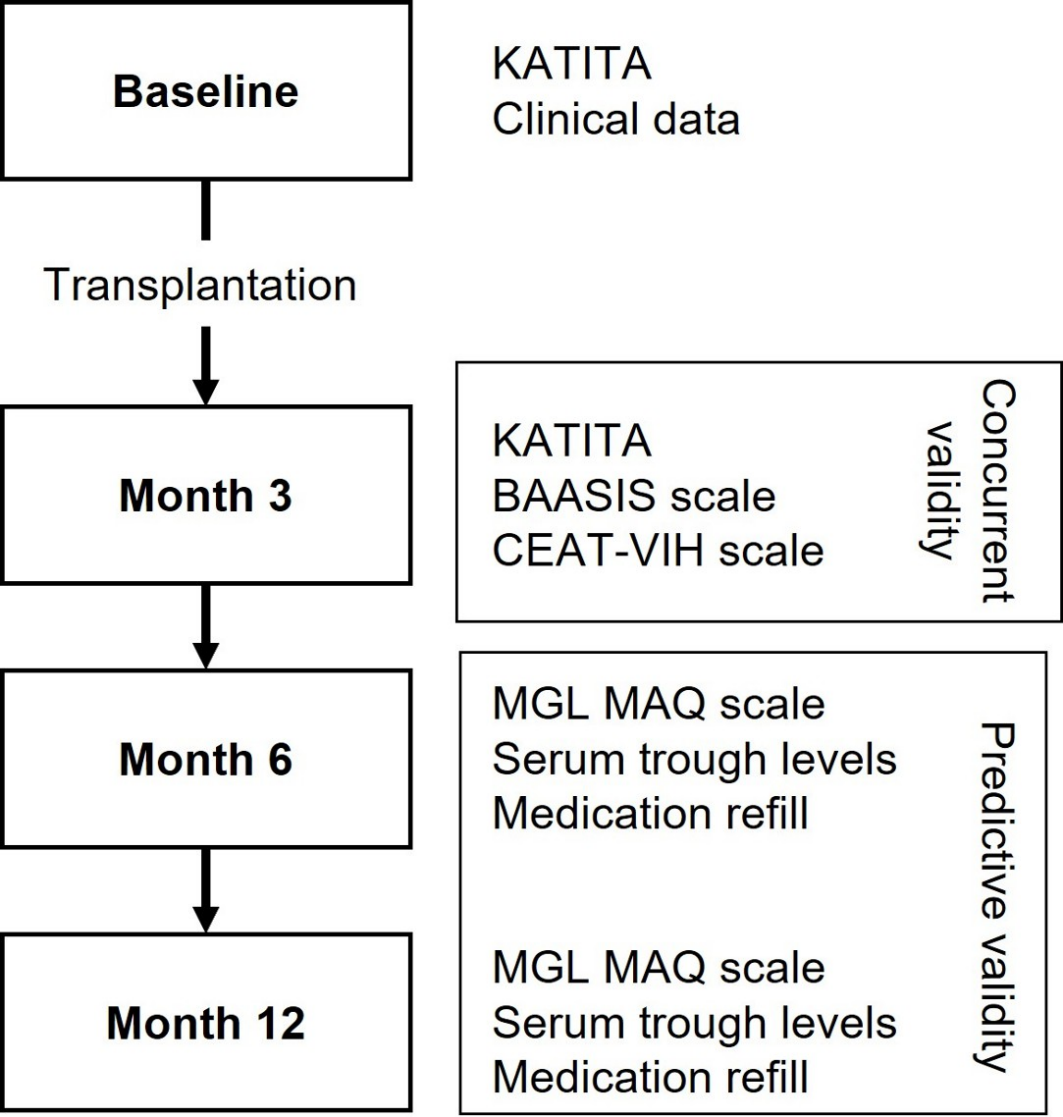
Diagram of the study design of the external validation study.

A prospective, open cohort study involving six kidney transplant centers in three state capitals will include consecutive patients over 18 years-old, of both sexes, who had just been scheduled for kidney transplantation from either a deceased or living donor. Patients will be excluded if they are hospitalized, planned for retransplant, recipients of additional non-kidney transplant, illiterate or with impaired cognition. As shown in Fig 3, the patients will be followed-up for 12 months after kidney transplant.

At the pre-transplantation, baseline evaluation, demographic and clinical data will be collected, and the KATITA questionnaire will be administered. The questionnaire data will be used for the assessment of predictive validity and of structural validity, which will evaluate whether the factor structure of the KATITA questionnaire is reproduced in this different population. The timing of kidney transplantation after the baseline visit will be variable but expected to be within a short time lag. The next study visit will be at 3 months post-transplant and its purpose will be to assess the concurrent validity of the questionnaire. Concurrent validity is demonstrated by shown a high correlation of the scores of the test scale with those of a validated scale measuring the same or a closely related construct. However, there is no available scale measuring predisposition to medication non-adherence in kidney transplant patients in the pre-transplant setting. To overcome this difficulty and to still obtain a measure of concurrent validity, in addition to the re-administration of the KATITA questionnaire the following two scales were selected to be compared with KATITA at three months post-transplant. The Basel Assessment of Adherence to Immunosuppressive Medication Scale (BAASIS), a widely used questionnaire for assessing adherence to immunosuppressive drugs in adult and adolescent post-transplant recipients, consists of four questions inquiring the patient about the past occurrence of medication non-adherence episodes, two being about the frequency of missed medications doses, one on non-compliance with medication timing, and one asking about having reduced medication doses. Each question has six possible responses, from never (0 points) to everyday (5 points), and thus the questionnaire scores range from 0 to 20, with a score greater than zero classifying the patient as medication non-adherent. The CEAT-VIH scale [29] will also be applied and used as another reference against which the KATITA scores will be compared. This scale was developed to assess medication adherence of persons living with HIV infection and, in addition to questions about non-adherence episodes, includes questions assessing patients’ behavior and beliefs towards their medication. It is a 20-item scale forming a score between 18 and 92, lower scores indicating lower adherence to medication. This scale was selected because it produces an ordinal score measuring the degree of medication non-adherence, which is a distinctive feature from virtually all other medication adherence instruments that only discriminate between adherents and non-adherents, but also because HIV therapy has the same requirements as immunosuppressive therapy in transplant patients regarding strict adherence to the prescribed frequency, dosage, and timing of medication administration. The month 3 study visit will also offer the opportunity of testing KATITA’s sensitivity to change, the ability to respond to a change in state regardless of its clinical relevance to the patient or the clinician, by evaluating whether there was variation in the scale scores after patient exposure to immunosuppressant medication since the pre-transplant evaluation.

The following study visits, at months 6 and 12 post-transplant, will be for evaluation of predictive validity. Predictive validity, the second component of criterion validity, is the ability of a questionnaire score to predict a future outcome, as expected by theory, which represents the criterion or gold standard. The main difference to concurrent validity is that in predictive validity the criterion is measured at a later time than the scale scores, while in concurrent validity both are measured at the same time. In this case, the criterion is obviously medication non-adherence. However, there is no single method for measurement of medication non-adherence that can be considered a gold standard. Methods for measurement of medication non-adherence may be divided into objective methods, including pill count, pharmacy refill records, serum level of immunosuppressants, and electronic monitoring of opening of pill bottles, and subjective methods, such as medication adherence scales, patient self-reporting, and physician impressions. A combination of methods is usually adopted in the evaluation of medication non-adherence to immunosuppressants and, in keeping with this approach, we will use three methods that independently discriminate medication adherent from non-adherent patients: patient self-report of non-adherence using the Morisky Green Levine Medication Assessment Questionnaire (MGL MAQ) [30], the trough serum levels (C_0_ levels) of immunosuppressants measured at the study visits, and reports on pharmacy refill of immunosuppressants. The MGL MAQ tool consists of four questions with a yes/no answer, two being about forgetfulness on taking medication, and two on behavior on taking medication. Patients will also be classified as medication non-adherents if trough serum levels of immunosuppressants measured at each 6- and 12-months study visit are lower than 4 ng/mL for tacrolimus [31], 4 ng/mL for sirolimus [32], 3 ng/mL for everolimus [33], and 50 ng/mL for ciclosporin [34]. All participating healthcare centers belong to the National Health System, which delivers immunosuppressant medication without costs to patients at a central pharmacy, making pharmacy refill records easily accessible for identification of patients who fail to get their medication and who will be considered, in this study, as medication non-adherents. A patient will be considered as non-adherent if being classified as non-adherent by any of the three methods at either the 6-month or the 12-month visit.

### Cross-cultural validity

In the fourth study phase, the KATITA questionnaire will be evaluated for cross-cultural generalizability and validity, that is, whether the questionnaire’s properties remain consistent and applicable when used in diverse cultural settings, and that the measurements remain valid and accurate across various cultural groups. The country selected for this study was Spain because it has had the world’s greater number of kidney donors per million population for over 30 years [35].

The cross-cultural adaptation will be performed according to the Guillemin protocol [36], starting with the Spanish translation of the KATITA questionnaire made by a native speaker of the language, which will be evaluated semantically by kidney transplant experts and patients to ensure conceptual equivalence. The Spanish text will be back translated into the original Portuguese language by a linguistic specialist without connection to the clinic and without knowledge of the original text. This process will continue iteratively until the final version of the instrument is agreed among the participants of the process. A multicenter prospective study will enroll Spanish patients consecutively observed in two kidney transplant centers and two hemodialysis clinics in Barcelona, Spain, with the same eligibility criteria of the external validity study: patients in the kidney transplant waiting list, of both sexes, over 18 years-old, excluding hospitalized patients, those planned for retransplant or recipients of additional non-kidney transplant, and illiterate patients or with impaired cognition. Demographic and clinical data will be recorded and patients will be asked to self-rate the translated questionnaire. A subsample of patients will be consecutively selected and invited to have the questionnaire re-administered one month later. This will conclude the KATITA’s study plan.

### Sample sizes

For the focus group, eight patients will be selected, which is within the recommended size for focus groups [37]. For the cognitive interviews, sample size will be determined by saturation, that is, when no new information is obtained from participants, but it expected to require no more than 10 patients. For the development of questionnaires measuring a latent construct using factor analysis, and for reliability assessment, there are neither theoretical foundations, nor formal methods of estimation of the required sample size, and the required numbers are based on rules of thumb suggested by several authors, with some recommending a sample size defined by a ratio of participants for each scale item, often 10:1 [38], while others propose a fixed number of participants, independent of the number of items, most commonly in the range of 200 to 300 subjects [39–42]. Accordingly, the sample size for this part of the study will be no less than 300 patients. For the test-retest reliability analysis, a sample size of 20 subjects would be sufficient to ensure 80% power with an alpha error of 0.05 to identify a correlation greater than 0.60, however such small sample will not provide adequate robustness to the results, so a minimum sample size of 60 patients was defined.

The sample size for the external validity study is determined by the requirements for the evaluation of the predictive validity of the KATITA questionnaire. Assuming that the area under the ROC curve (AUROC) of the questionnaire in the prediction of immunosuppressive medication non-adherence would be at least 0.75, and that the proportion of non-adherent patients in the first 12 months after transplant will be about 50%, a sample of 364 patients would estimate the AUROC with an error of ±0.10 with 95% confidence [43]. The same sample size will be used in confirmatory factor analysis for assessment of structural validity of the factorial model, scale sensitivity to change, and concurrent validity assessment.

For the cross-cultural generalizability and validation study, the sample size is defined as 5 patients per questionnaire item. This number was based on a systematic review of 114 articles reporting on the validation of psychometric scales, where the authors [44] concluded that there is no formal method to calculate the sample size to validate clinical questionnaires. In that review they reported that in 50% of the articles, the sample size was 10 subjects or less per questionnaire item. Other authors have proposed that the calculation of the sample size in a validation study of clinical questionnaires be determined based on the number of items in the questionnaire. Kline [45] proposed 2 subjects per variable, Hatcher [46] suggested 5 per item and a minimum of 100 subjects. Nunnally [38] suggested 10 per item and Pett et al. [47] 10–15 participants per item. Thus, the proposed proportion of 5 subjects per questionnaire item seems adequate for the purposes of this study. For the test-retest reliability assessment, again a subsample of 60 patients will be selected.

### Statistical analysis

STATA 18 statistical program (Stata Corp., College Station, TX, USA) will be used for statistical analysis. All tests will be two-tailed and results will be considered statistically significant when p<0.05.

### Questionnaire development

Exploratory factor analysis will be used for questionnaire development. Only those factors with an eigenvalue greater than 1, that is, factors that explain the variability in the scale more than a single item (Kaiser criterion), will be retained. If factor rotation is considered necessary to improve item selection, a varimax rotation, which is an orthogonal rotation, will be applied. Factor loadings less than 0.40 will be ignored. Items presenting a commonality significantly lower than the value observed for the remaining items will also be discarded. Pearson’s correlation will be used for item analysis and the following rules will be adopted for item reduction: inter-item correlations should not be greater than 0.70, which may indicate redundancy, neither less than 0.30, which suggests the item is measuring something different; item-test correlation, the correlation of each item with the scale score, should be high (>0.30), otherwise the item is probably measuring something different; item-rest correlation, the correlation of each item with the score formed with the remaining items, should be moderately low (between 0.30 and 0.70), otherwise the item may be redundant [28]. If a multidimensional scale is obtained, the item-to-own dimension correlation, the correlation of each item with the total score of its own subscale, should be high (>0.30). The assumption of independence of the factors will be tested with an inter-dimension correlation matrix and will be discarded if correlations are moderately high (>0.60).

### Internal validity assessment

The internal consistency reliability of the final version of the KATITA questionnaire will be evaluated with Cronbach’s alpha for the full scale and for each dimension if a multidimensional scale is obtained, with 95% confidence intervals estimated by bootstrapping. It is generally accepted that for a scale to have clinical application its Cronbach’s alpha must be above 0.80. This statistic will also be used to refine the questionnaire: leaving-one-out Cronbach’s alpha, a Cronbach’s alpha computed excluding one item in turn, should be somewhat smaller than the scale’s Cronbach alpha, otherwise it means that the left-out item is not contributing to the internal consistency reliability. Should factor analysis point to a multidimensional scale, Cronbach’s alphas will be computed for each subscale as well. The same final version of KATITA will be assessed for test-retest reliability using the two-way mixed-effects model, absolute agreement, intraclass correlation coefficient. The generally accepted cutoffs for the coefficient will be adopted: less than 0.50 (poor reliability), 0.50–0.75 (moderate reliability), 0.75–0.90 (good reliability) and greater than 0.90 (excellent reliability). For the evaluation of convergent validity with differentiation by known groups, the Wilcoxon-Mann-Whitney ranksum test will be used to compare KATITA scores between levels of risk factors for non-adherence to immunotherapy medication.

#### External validity assessment

For the analysis of the scale sensitivity to change, the time interval between baseline and transplantation will be presented by the median and interquartile range, and the change will be tested with the Wilcoxon signed rank test comparing baseline to 3-month scores. The magnitude of the change will be interpreted using the standardized response mean (SRM), which is the mean difference between baseline and 3-month post-transplant scores divided by its standard deviation [48], as moderate if SRM>0.5, or large if SRM>0.8. For the evaluation of concurrent validity, Spearman’s rho will be used to estimate the correlation between KATITA scores and the scores of the BAASIS and the CEAP-VIH scales, and the Wilcoxon-Mann-Whitney ranksum test will be used to test the difference in KATITA scores between adherent and non-adherent according to BAASIS. For the evaluation of the predictive validity of the KATITA questionnaire, the AUROC, sensitivity, specificity, positive predictive value and negative predictive value, and corresponding 95% confidence intervals, will be computed for pre-transplant KATITA scores. Structural equation modeling will be used for confirmatory factor analysis, with goodness of fit evaluated with the chi-square test/degrees of freedom statistic, Root Mean Square Error of Approximation (RMSEA), Comparative Fit Index (CFI), Tucker-Lewis index, standardized root mean squared residual and coefficient of determination.

#### Cross-cultural validation

A confirmatory factor analysis using structural equation modeling will be conducted to test the factor structure. Internal consistency reliability will be assessed with Cronbach’s alpha. Test-retest reliability will be evaluated with the two-way mixed-effects model, absolute agreement, intraclass correlation coefficient.

## Dissemination

The results of this research will be published in peer-reviewed scientific journals and presented at scientific conferences. All data from this research will be made publicly available as supplementary files of the published papers. The validated scale and scoring instructions will be freely available.
